# Postural Education in School-Aged Populations: Development and Usability Evaluation of a Mobile Biofeedback App (EduBack)

**DOI:** 10.2196/79282

**Published:** 2026-02-26

**Authors:** Marco A García-Luna, Miguel García-Jaén, Daniel Ruiz-Fernández, Carmen Manchado, Juan M Cortell-Tormo

**Affiliations:** 1 Department of General and Specific Didactics, Faculty of Education, University of Alicante, C/ Aeroplano, s/n, San Vicente del Raspeig, 03690, Spain, 34 965903400 ext 2142; 2 Department of Computer Science and Technology, University of Alicante, San Vicente del Raspeig, Spain

**Keywords:** postural education, wearable technology, inertial sensors, mobile health, biofeedback, usability evaluation, motor learning, app development, mobile health innovation

## Abstract

**Background:**

Postural education is crucial during childhood and adolescence, yet traditional approaches often lack engaging tools that promote awareness and behavioral change. Wearable technologies and real-time biofeedback systems offer new opportunities to support postural learning through immediate, embodied feedback. However, most existing systems focus on clinical rehabilitation, with few designed specifically for educational use.

**Objective:**

This study aimed to design, develop, and evaluate the usability and technical performance of EduBack, a mobile app that delivers real-time lumbar posture biofeedback through inertial sensors, with a specific focus on educational settings such as schools and physical education environments.

**Methods:**

EduBack was developed using Kotlin (JetBrains) for Android OS (Google; version 8.0 and above) and integrates with 2 inertial measurement units via Bluetooth (2.4 GHz). The app provides visual biofeedback through a dynamic interface showing a virtual spine, corrective messages, and a color-coded alignment bar. The usability evaluation involved 24 undergraduate students (mean age 21.4, SD 1.8 y) who used the app in a controlled session. Participants completed the system usability scale and open-ended qualitative feedback questions. Technical performance data were collected from system logs, latency measurements, and received signal strength indicator values to assess connection stability and sensor-to-app communication.

**Results:**

The average system usability scale score was 83.5 (SD 8.7), indicating excellent usability. Participants reported the interface to be intuitive, the biofeedback visualization clear, and the posture information easy to interpret. Qualitative responses highlighted the app’s potential to support postural awareness and motor learning, especially in school-aged populations. From a technical perspective, the system demonstrated robust performance: mean data transfer latency was approximately 120 milliseconds, with less than 1% packet loss across sessions. Received signal strength indicator values consistently remained within the optimal signal range, confirming stable Bluetooth connectivity. All session data were successfully stored and exported without errors. The real-time posture tracking displayed on the app closely matched raw sensor data, ensuring fidelity in feedback.

**Conclusions:**

EduBack is a usable and technically stable mobile app designed to support postural education through wearable sensors and real-time biofeedback. Its user-friendly interface and reliable data transmission make it well-suited for use in schools and educational programs targeting postural health. The app fills a gap in the mobile health field by offering a preventive, educational tool rather than a clinical one. Future research should explore its application in younger populations, integration into physical education curricula, and long-term effects on postural behavior and motor skill acquisition.

## Introduction

Musculoskeletal disorders represent one of the most prevalent health problems across different age groups and populations and are currently recognized as a major global public health concern [1,2]. Among these, low back pain (LBP) is particularly significant, as it is one of the most widespread conditions [3,4], surpassing complaints in other spinal regions such as the cervical or thoracic spine [4,5]. Its global incidence has increased by approximately 55% over the last 3 decades, making it the leading cause of work-related disability today [3-9]. Current estimates suggest that by 2050, nearly 850 million people worldwide will suffer from LBP, representing a 36% increase from 2020 levels [4].

Unfortunately, children and adolescents are not exempt from this musculoskeletal condition [10,11]. The lifetime prevalence of LBP by ages 10‐12 years is approximately 20%, increasing significantly during adolescence and reaching rates comparable to adults—up to 70% by late adolescence [12,13]. Several studies have highlighted the upward trend in LBP prevalence among adolescents over the last 2 decades [1,14,15].

LBP is considered a multifactorial syndrome, influenced by a range of biological, biomechanical, psychological, emotional, behavioral, and environmental factors [16-21]. Among the modifiable risk factors, poor postural hygiene, obesity, improper lifting techniques, prolonged sedentary behavior, and low levels of daily physical activity have been identified [5,7,16,20-23]. Although multidisciplinary approaches are often recommended for LBP prevention and treatment [24], there is broad consensus that physical exercise combined with posture-focused educational programs is one of the most effective strategies for reducing both the incidence and severity of LBP [3,25-27].

Numerous researchers and international organizations have advocated for posture education and its integration into school-based programs as a strategy to prevent both present and future LBP [28-39]. This may be due to the strong potential of educational institutions to equip children and adolescents with the knowledge and skills necessary to preserve and improve their health [39,40]. Indeed, one of the most powerful tools to prevent back pain—or at least reduce its frequency and severity—is the implementation of educational interventions that promote long-term acquisition of postural knowledge and healthy habits [39,41].

Technology plays a pivotal role in modern education and has demonstrated clear benefits in enhancing student learning outcomes [42-44]. In this context, biofeedback has emerged as an efficient and effective tool for enhancing postural awareness and promoting corrective behavior in diverse activities and settings [45-48]. Biofeedback is a technique that monitors and quantifies physiological or biomechanical parameters and converts them into real-time visual, auditory, or tactile signals [49,50]. Technological advances have enabled the integration of biofeedback into wearable systems, which offer advantages such as portability, lightweight, low cost, and energy efficiency [51-53].

Despite these advances, wearable tools specifically designed to provide postural biofeedback in educational settings remain limited [45,54]. This scarcity is largely due to the recent emergence of such technologies, as well as the lack of published studies evaluating their implementation and effectiveness in schools. Although some posture-monitoring and correction devices exist, most are designed for clinical or sports use and are poorly adapted for educational contexts. As a result, further research is needed to explore the role of these technologies in teaching and learning healthy postural habits. In particular, such research is envisioned for children and adolescents in both primary and secondary school settings, where early intervention is most relevant for long-term musculoskeletal health.

Therefore, the objective of this study was to develop and preliminarily evaluate EduBack, a mobile app based on inertial sensors and real-time biofeedback. Specifically, this study aimed to assess the usability and technical performance of the system as a first step toward its long-term educational goal of supporting the learning and internalization of correct lumbar posture in school settings. The study followed a descriptive technological development design with an embedded user evaluation, combining the development of the EduBack system with a preliminary assessment of its usability and technical reliability.

## Methods

### Study Design

This study followed a cross-sectional descriptive technological development design with an embedded user evaluation (mixed methods approach). Detailed methods are presented in the following subsections.

### Participants

Although the long-term target population of EduBack is children and adolescents, university students were intentionally selected for this preliminary evaluation to ensure feasibility and technical stability before adapting the system to younger users. Therefore, a convenience sample of 24 undergraduate students (mean age: 21.4, SD 1.8 y; n=10, 42% female participants) from the Faculty of Education was recruited. Inclusion criteria were: (i) no known musculoskeletal or neurological conditions affecting posture and (ii) basic familiarity with smartphone use.

### Ethical Considerations

All participants provided written informed consent after receiving detailed information about the study procedures, potential risks, and their right to withdraw at any time without penalty. This study was approved by the Ethics Committee of the University of Alicante (reference number UA-2023-11-16) and conducted in accordance with institutional guidelines and the principles of the Declaration of Helsinki.

All data were anonymized prior to analysis, and no identifying information was collected or stored. Privacy and confidentiality were ensured throughout the study, with data accessible only to the research team. Participants did not receive financial or material compensation for their involvement. The sample consisted of adult undergraduate students, who are not considered a vulnerable population.

### Tools and Instruments

#### System Conceptualization

Based on a review of the literature on wearable tools for postural education [51-53], design principles for educational systems aimed at postural training [36,40,55], usability evaluations of similar health technologies [56,57] and user reviews of commercially available mobile apps for postural correction (Google Play and Apple App Store), 3 core features were defined to build an effective mobile educational system for postural hygiene and training: (i) real-time biofeedback of lumbar posture using inertial sensors, providing immediate visual feedback to raise awareness and enable posture correction; (ii) local data recording and storage for long-term postural monitoring; and (iii) personalized recommendations based on the user’s postural patterns, including suggestions, adaptations, or corrective cues.

Furthermore, for the system to be suitable for long-term educational use, we established the following usability requirements: (i) the interface must be intuitive and user-friendly, requiring no prior technical knowledge from students and (ii) both data collection and postural feedback must be automated and seamlessly integrated into activities to avoid interfering with the learning process.

#### App Development

During the initial development phase of the EduBack system, a list of key parameters for real-time postural monitoring and biofeedback was formulated. Due to the lack of existing solutions that met our specifications—particularly apps offering intuitive and real-time visualization of lumbar posture in educational settings—we developed a dedicated mobile app to effectively collect and display this information.

Technically, the EduBack system is composed of two primary components: (i) a mobile app designed for local data collection and real-time visual feedback, enabling students to autonomously and effectively monitor their posture and (ii) an inertial measurement unit (IMU) system, consisting of 2 IMU devices placed on the user’s lumbar spine. These devices connect via Bluetooth to a smartphone or tablet running the EduBack app and are responsible for capturing data on spinal alignment and movement.

In addition, the EduBack app integrates a computer vision module (MoveNet) to provide a simplified visual representation of the user’s skeleton. This representation is based on 17 anatomical reference points (head, shoulders, elbows, wrists, hips, knees, and ankles), detected in real time through the smartphone or tablet camera and displayed using Canvas. The purpose of this visualization is to complement the inertial sensor data with intuitive feedback, enhancing user awareness of posture during educational tasks. Importantly, this skeletal visualization was intentionally designed as a set of abstract points rather than a full video image, in order to respect user privacy. This approach is particularly relevant for the intended target population of children and adolescents, as it allows external recording of postural tasks by another person without displaying identifiable visual features of the participant.

#### Overview of the EduBack System

**Figure 1.**
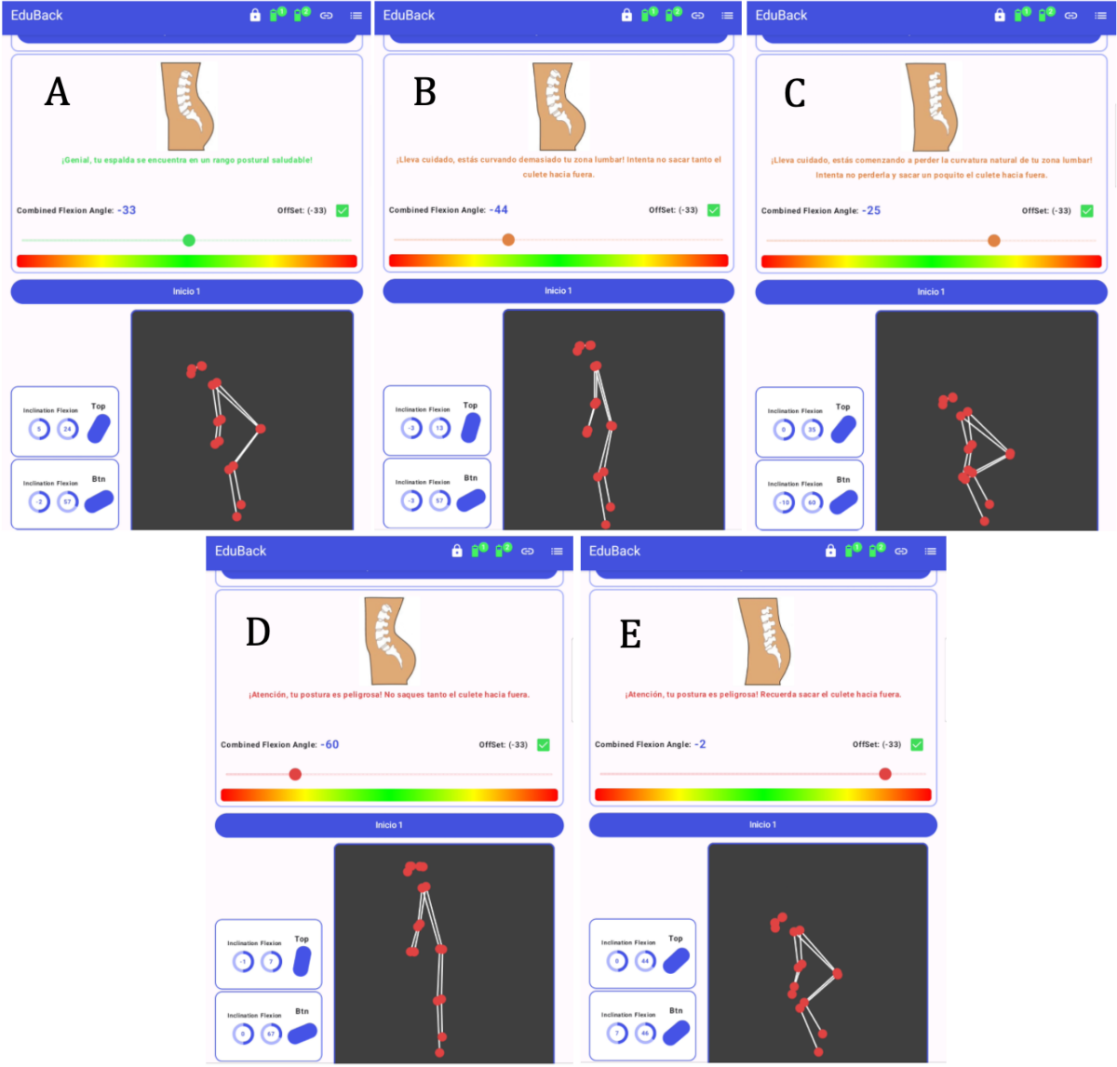
Illustrative examples of the EduBack app functioning and its biofeedback indicators for lumbar posture. Panel A shows the neutral lumbar lordosis, which refers to the natural inward curvature of the lumbar spine in standing posture. Panels B and C represent intermediate deviations from this neutral posture. Panel D illustrates lumbar hyperlordosis, defined as an excessive inward curvature of the lumbar spine. Panel E illustrates lumbar kyphosis, defined as an abnormal outward curvature of the lumbar spine in the lumbar region. Together, these examples demonstrate how the EduBack app provides real-time visual feedback on different postural states.

**Figure 2.**
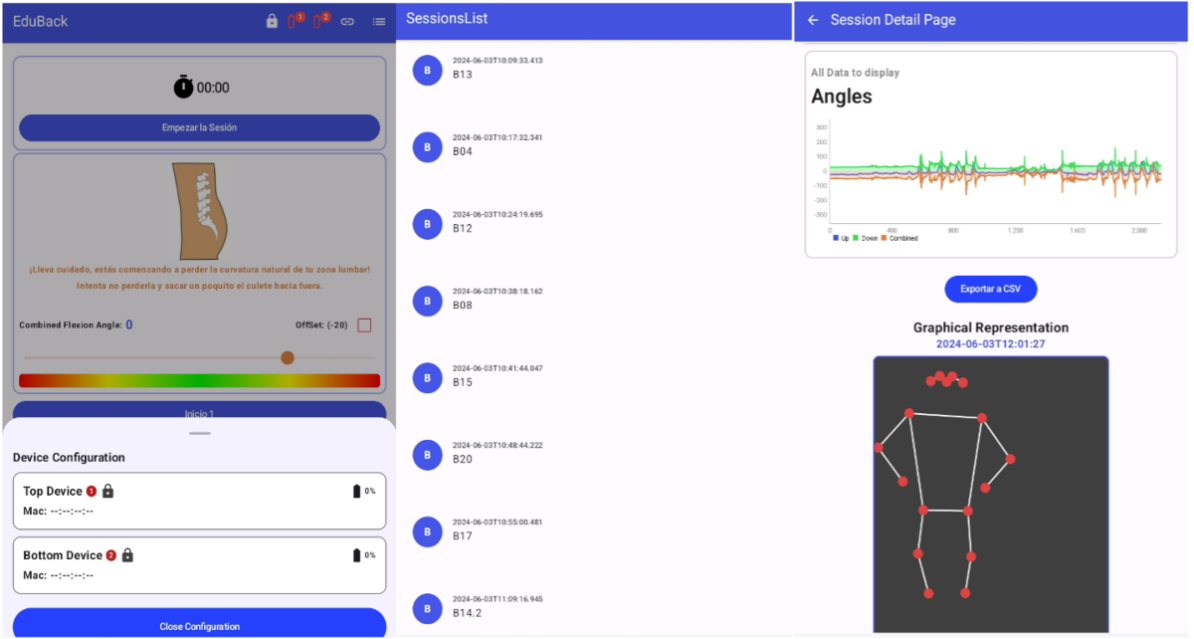
Main screens of the EduBack mobile app. The Home screen (left) displays the sensor status (connection and inclination), a real-time skeleton visualization based on 17 anatomical points detected by the device camera, and integrated biofeedback including a spinal visualization, color bar, and dynamic dot indicating posture. The List screen (center) provides an overview of recorded sessions. The Detail screen (right) shows ratings and key information for each recorded session, allowing users to review their performance and feedback.

EduBack is a mobile app developed using Kotlin for devices running Android OS 8 and above. It features three main screens: Home, List, and Detail. The Home screen basically displays: (i) the sensor status (connection, inclination, etc); (ii) a real-time visual representation of 17 points, based on Canvas and MoveNet, showing the human skeleton detected by the smartphone or tablet camera; and (iii) the integrated biofeedback that includes a spinal visualization with associated messages, a color bar, and a dynamic dot that moves along the bar according to the user’s posture. The List screen provides an overview of recorded sessions. In the Detail screen, users can view ratings and key details for each recorded session. Figure 1 displays the 3 main screens of the app, while Figure 2 illustrates some graphical representations of the EduBack app’s functionality and its biofeedback, such as when lifting a heavy object from the ground.

The design of the EduBack interface was informed by principles of motor learning, instructional design, and behavior change. Systematic reviews and meta-analyses have consistently shown that color-coded visual cues and dynamic prompts enhance attentional focus, facilitate error correction, and support self-regulated learning in motor tasks [58,59]. Similarly, systematic reviews in instructional design highlight the pedagogical value of visual displays for improving comprehension and engagement [60]. Finally, reviews of digital behavior change interventions confirm that immediate, intuitive feedback is a key driver of adherence and habit formation [61,62]. These findings provide a strong theoretical and empirical foundation for the interface design choices implemented in EduBack.

**Figure 3.**
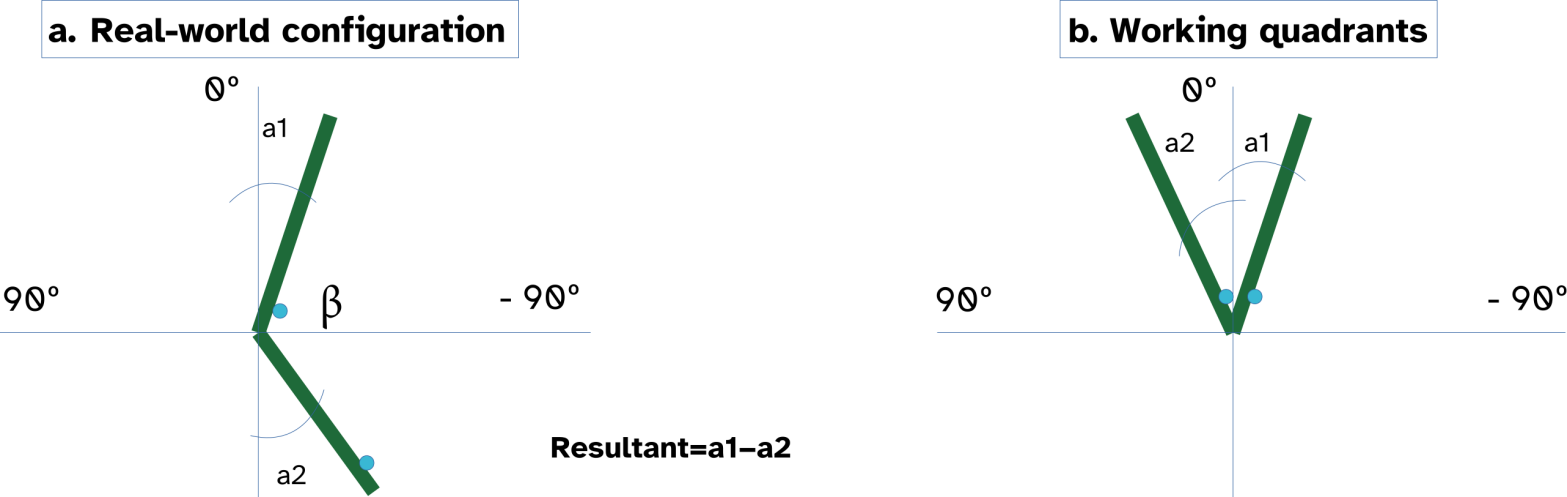
Representation of the lumbar angle calculation using 2 inertial measurement units. Each sensor (placed at L1 and S1) provides an inclination relative to the vertical axis. By combining these values, the relative lumbar angle (β) is obtained, with the sign indicating orientation. Blue points represent the inertial measurement unit LEDs, showing sensor orientation.

Figure 3 illustrates how the lumbar angle was estimated from the inclinations of the 2 IMUs placed at L1 and S1. Each sensor provided a flexion angle relative to the vertical axis (A1 and A2). In practice, these angles are calculated in opposite quadrants, with opposite signs. By subtracting them, the resultant angle corresponds to the relative inclination between the 2 sensors, with the sign indicating the orientation direction. The lumbar angle β was then obtained as 180 minus this resultant value. This computational approach has been previously validated in García-Luna et al [63], where IMU-derived pelvic and lumbar tilt angles showed high agreement with a 3D optical motion capture system, considered the gold standard for kinematic assessment.

#### Data Processing Software

The mobile app connects to the inertial sensors using Bluetooth Low Energy, a protocol that supports low-latency communication. Once paired, the device transmits data via notifications, which are received by the smartphone. Internally, the data—originally in byte format—is decoded using bitwise operations and mathematical transformations to convert hexadecimal values into decimal format, allowing for accurate on-screen visualization.

While receiving the data, the app stores it in a local database to enable later retrieval and export in CSV format. This export process transforms the locally stored tabular data into rows and columns, a common practice in this type of system. Storage capacity depends on the available device memory; however, modern smartphones typically do not present limitations in this regard. The app uses a secure local database implemented with Room (Android persistence library) to ensure reliable record keeping.

At the beginning of each session, the user must connect the sensors, triggering real-time data transmission and visualization. During the session, data is temporarily stored in cache memory; once the session ends, the data is transferred to the local database. This process ensures data persistence and prevents information loss if the app is restarted.

#### Inertial Sensors

The inertial sensors used were Ergotex IMUs (JVTech Solutions), designed specifically for spinal posture monitoring. Each device operates at a frequency of 20 Hz and is equipped with a triaxial gyroscope (±1000 deg/s) and triaxial accelerometer (±2 g), enclosed in a compact unit measuring 21×10 mm and weighing only 8 grams. These sensors record acceleration across 3 axes and transmit data in real time via Bluetooth (2.4 GHz) to a mobile device running the EduBack app.

To ensure optimal performance, each Ergotex IMU integrates the ICM-20602 MEMS MotionTracking sensor (TDK Corp), known for its high accuracy and reliability. This sensor offers a gyroscope sensitivity error within ±1% and gyroscope noise levels of ±4 mdeg/s/√Hz, while accelerometer noise is maintained at 100 μg/√Hz. Additionally, its integration with a 1K-byte first-in, first-out buffer reduces serial bus traffic, enhancing measurement stability and energy efficiency.

The system includes a battery connected to a regulated power supply to ensure a stable voltage output. A microprocessor collects the raw data from the accelerometer and gyroscope through the serial peripheral interface bus and sends the processed information to a radio frequency module for wireless transmission. This system allows for efficient data flow and ensures the precision and reliability of postural data collection.

Importantly, the concurrent validity of these inertial sensors for estimating lumbar and pelvic tilt angles has been previously established against a 3D optical motion capture system, considered the gold standard for kinematic assessment. García-Luna et al [63] reported minimal systematic error (0.08° overall) and high agreement between IMU-derived angles and optical measurements, with correlation coefficients exceeding 0.90 and mean absolute errors around 1°. Bland-Altman analyses confirmed that most data points fell within ±2° limits of agreement, supporting the accuracy of the Ergotex IMUs for sagittal pelvic tilt measurement in both static and dynamic positions. These findings validate the suitability of the sensors for postural monitoring in educational contexts and justify their use in the present study.

#### Usability Assessment Instrument

To assess the perceived usability of the EduBack app, participants completed the Spanish version of the system usability scale (SUS) [64] immediately after the testing session. The SUS is a widely used, validated 10-item questionnaire that provides a global view of subjective usability [65,66]. Each item is scored on a 5-point Likert scale ranging from “strongly disagree” to “strongly agree.” The total score ranges from 0 to 100, with higher scores indicating better usability. The SUS was selected due to its brevity, ease of application, and frequent use in digital health evaluations, making it an appropriate benchmark for assessing mobile health-related apps [67-69].

In addition to SUS, qualitative comments on educational value (biofeedback clarity, motor awareness, and school applicability) were collected post-session to complement usability scores.

### Procedure

**Figure 4.**
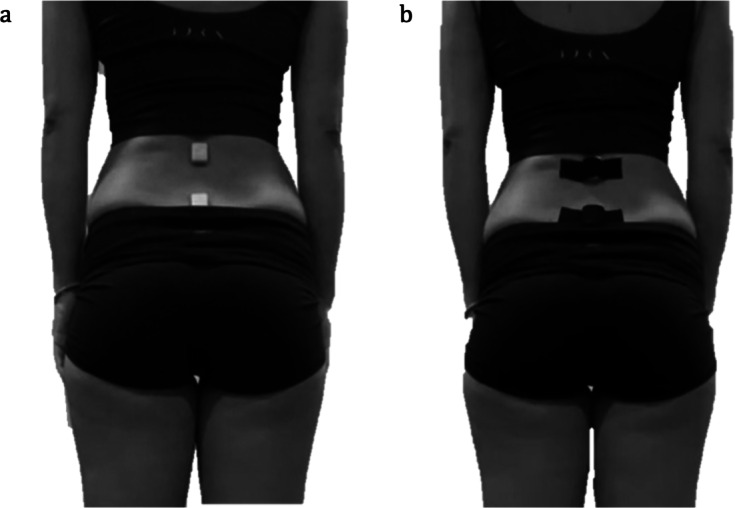
. Placement of the inertial sensors over the spinous processes of L1 and S1. (A) Sensors were initially attached to the skin using hypoallergenic double-sided tape. (B) Sensors were subsequently secured with kinesio tape to ensure stability during the 30-minute postural task protocol.

Participants were individually invited to a simulated laboratory setting replicating a postural education session. Sensor placement over the spinous processes of L1 and S1 (Figure 4) was performed directly by a trained researcher to ensure consistency and reliability of the instrumentation. Likewise, all instructions regarding the use of the EduBack app and the inertial sensors were provided orally by the researcher during the session, with no written guide, to guarantee standardized guidance across participants. Each participant then used the EduBack app on a standardized tablet provided in the laboratory during a guided 30-minute session that included a predefined sequence of postural tasks. The protocol included anterior and posterior pelvic tilts in both standing and seated positions, forward and backward trunk inclinations, picking up an object from the floor, and repeated sit-to-stand transitions from chairs of different heights, in addition to maintaining upright and seated postures. All participants performed the same tasks in the same order under standardized instructions. Sessions were conducted individually, with each participant completing the protocol one by one under controlled laboratory conditions. During the session, real-time feedback was displayed on the app. After completing the session, participants were asked to evaluate the app’s usability using a validated questionnaire. In addition, brief qualitative comments were collected regarding the perceived educational value of the biofeedback (clarity, motor awareness, and applicability in school contexts), complementing the quantitative usability scores.

### Data Analysis

Descriptive statistics were used to analyze the usability data. SUS scores were calculated according to standard methods [65], and summarized using means, SDs, and score ranges. Technical performance data were collected through internal logs generated by the EduBack app, including latency (sensor-to-screen transfer time), Bluetooth connection stability, and data packet loss rate. Signal quality was additionally assessed using the Received Signal Strength Indicator, a standard metric for evaluating the strength of wireless signals. Based on predefined thresholds, signal quality was classified as optimal (−50 to −70 dBm), moderate (−70 to −80 dBm), or poor (below −93 dBm), with all sessions falling within the optimal range. These metrics were used to evaluate the robustness of the hardware-software integration and the reliability of real-time postural monitoring. Analyses were conducted using SPSS Statistics (version 27; IBM Corp). Qualitative feedback was collected in the form of brief participant comments at the end of each session and reviewed descriptively to complement the quantitative usability scores.

## Results

### System Usability

The usability of the EduBack app was assessed using the SUS (Spanish version), a widely validated instrument for evaluating user satisfaction and perceived system usability. The overall mean SUS score obtained was 83.5 (SD 8.7), which corresponds to an “excellent” rating according to established interpretation thresholds [66]. This result places the system well above the average usability benchmark of 68, indicating a high level of user satisfaction and ease of use.

A closer inspection of individual SUS items revealed that the system was particularly well-received in terms of learnability and confidence. For example: (1) 90% (18/20) of participants “strongly agreed” or “agreed” that they felt confident using the system; (2) 85% (17/20) considered that most people would learn to use the app very quickly; and (3) 90% (18/20) agreed that the app was easy to use and that its functionalities were well integrated.

Items related to system complexity and the need for technical support also received favorable evaluations: 90% (18/20) of users reported that they did not require technical assistance to use the app, and 85% (17/20) strongly disagreed with the statement that the system was unnecessarily complex.

Despite these high scores, certain aspects showed slightly more variability. While still favorable, the lowest-rated item was related to the consistency of the interface, where 70% (14/20) users “strongly agreed” or “agreed” that the various system functions were well integrated, and 20% (4/20) selected a neutral response. This suggests a potential area for further refinement in terms of user interface design consistency and alignment across different screens or tasks. No participants rated the system as cumbersome or difficult to use, and no significant usability issues were reported during the testing sessions.

A detailed breakdown of responses to each SUS item is provided in Table 1, which highlights item-level agreement rates and helps pinpoint specific strengths and areas for improvement.

**Table 1.** Participant responses to individual SUS^a^ items (N=20).

SUS item	Statement (paraphrased)	Agree/strongly agree, n (%)	Neutral, n (%)	Disagree/strongly disagree, n (%)
1	I would use this system frequently	17 (85)	2 (10)	1 (5)
2	The system is unnecessarily complex	1 (5)	2 (10)	17 (85)
3	The system was easy to use	18 (90)	2 (10)	0 (0)
4	I would need help from a technical person	1 (5)	1 (5)	18 (90)
5	The functions were well integrated	14 (70)	4 (20)	2 (10)
6	The system was inconsistent	2 (10)	3 (15)	15 (75)
7	Most people would learn to use it quickly	17 (85)	2 (10)	1 (5)
8	The system is cumbersome to use	0 (0)	1 (5)	19 (95)
9	I felt confident using the system	18 (90)	2 (10)	0 (0)
10	I needed to learn a lot before using the system	1 (5)	2 (10)	17 (85)

a SUS: system usability scale.

### Technical Performance

The Bluetooth connectivity between the IMU sensors and the EduBack app remained consistently stable throughout all test sessions. System performance logs indicated an average latency of 120 milliseconds for sensor-to-screen data transfer, ensuring a responsive user experience. The rate of data packet loss was minimal, remaining below 1% across all sessions, and no failures in data storage or export were recorded.

Received signal strength indicator values monitored during the sessions consistently remained within the optimal range, confirming stable wireless communication between the sensors and the mobile device as defined by technical thresholds.

Real-time lumbar posture tracking was accurately displayed within the app’s biofeedback interface, as confirmed by synchronized screenshots and analysis of the raw data logs. These results support the technical reliability of the EduBack system for continuous, real-time postural monitoring in educational settings.

### Qualitative Feedback

Qualitative feedback collected during the usability evaluation provided additional insights beyond the SUS scores. Participants highlighted that the real-time biofeedback display, with its color-coded indicators and dynamic corrective prompts, enhanced their understanding of lumbar movement and posture control. Several students emphasized that the app facilitated motor awareness and contributed to acquiring correct lumbar mobilization patterns, supporting functional learning and the transfer of healthy postural habits to everyday activities. Importantly, participants suggested that EduBack could be particularly beneficial in school settings, especially for children and adolescents, where early postural education is essential for prevention and long-term musculoskeletal health. These comments illustrate the perceived educational value of the system and its potential applicability in real-world contexts.

## Discussion

### Principal Findings

This study presents the development process (described in the Methods) and reports the preliminary evaluation of EduBack, an innovative mobile app designed to deliver real-time postural biofeedback through wearable IMU sensors. The primary goal of the present study was to evaluate usability and technical reliability, establishing feasibility for future educational apps. While the long-term aim of EduBack is to facilitate postural education and the internalization of correct lumbar posture, these educational outcomes were not directly assessed here and remain directions for future research.

### System Usability

From a usability perspective, the results demonstrated that EduBack achieved high acceptance among participants, with SUS scores indicating strong usability. Feedback from undergraduate students was notably positive. Participants appreciated the intuitive interface, particularly the biofeedback visualization that included dynamic color bars, real-time vertebral tracking, and corrective prompts. These findings suggest that the interface design and feedback mechanisms are transferable to educational contexts, although future studies should confirm this in younger populations. This type of immediate, embodied feedback appears to support postural self-awareness—a key aspect of motor learning—and may foster metacognitive reflection about posture, which is rarely achieved through traditional instruction alone. Unlike clinical systems where usability is often compromised by multisensor burden or complex interfaces, EduBack’s simplicity may represent an advantage for adoption in schools. These outcomes align with prior findings on the effectiveness of biofeedback systems in physical rehabilitation and posture correction [45-48], but extend them by highlighting usability in an explicitly educational context.

### Technical Performance

Regarding technical performance, the system proved reliable under real-time conditions. The results confirmed that the system performs with low latency and minimal data loss during sensor-to-screen transmission. Participants experienced stable Bluetooth connectivity and consistent visual feedback, which suggests that the system is well-suited for educational environments that demand real-time interaction and reliability. Additionally, session data were securely stored and successfully exported, which is essential for both instructional follow-up and research use. It should be noted that in this context, “reliability” refers to technical robustness rather than psychometric reliability of the instrument. Psychometric reliability (eg, test-retest or interrater consistency) was not assessed in this preliminary study and remains an important direction for future validation. Prior studies have reported frequent technical challenges in wearable systems (eg, unstable wireless connections and battery limitations), which often hinder long-term use [70,71]. By demonstrating stable connectivity and efficient data handling, EduBack addresses some of these recurring barriers, although further testing in uncontrolled environments (eg, crowded classrooms) and formal reliability analysis are needed to confirm its robustness and measurement consistency.

### Qualitative Feedback

Qualitative feedback provided complementary insights into the educational value of EduBack. EduBack is distinguished from other wearable-based systems by its explicit focus on postural education rather than clinical intervention. Most prior research in this field has focused on rehabilitation in patients with chronic back pain, scoliosis, or musculoskeletal disorders [45,53,56]. Clinical and occupational apps of wearable posture systems commonly collect user-level information (eg, perceived usefulness, convenience, comfort, and practicality) alongside objective kinematic outcomes. Reviews consistently highlight real-time corrective feedback as promising, but also report heterogeneous and often limited documentation of user preferences and compliance, as well as recurring usability challenges (multisensor burden, attachment or removal, wearability over time, wireless stability, battery or power management) that impact acceptance and scalability. In contrast, the feedback gathered in the present study was explicitly educational: participants emphasized biofeedback clarity, enhanced motor awareness, and applicability in school settings—underscoring shared mechanisms (feedback-guided posture correction) but different aims (therapeutic adherence and symptom management versus prevention and movement literacy in educational contexts). This preliminary evidence of educational value, although not yet linked to measurable learning outcomes, highlights the potential of EduBack to bridge the gap between technological feasibility and pedagogical impact.

Building on this distinction, EduBack is designed for educational training, where the goal is not treatment but awareness, prevention, and acquisition of correct motor habits. This is particularly relevant in school-based educational contexts, where early intervention in postural habits can play a key role in long-term musculoskeletal health. By offering an engaging and interactive biofeedback experience, EduBack could facilitate motor learning of lumbar mobilization and may encourage the correct execution of daily movements from an early age. While the app may also support training in higher education or preprofessional programs as a secondary use case, its primary value lies in promoting postural awareness and self-regulation among children and adolescents in primary and secondary education settings.

Although EduBack was designed primarily for educational settings, several students highlighted its potential for use in clinical teaching environments, suggesting that the system could also help bridge the gap between academic learning and applied health care practice. The visualization of spinal posture, when combined with immediate feedback, may reinforce theoretical content from lectures and provide a meaningful learning experience. This aligns with pedagogical models that emphasize experiential learning and self-regulation in motor skill acquisition [72-74]. Future iterations of EduBack will incorporate gamification strategies (eg, rewards and interactive challenges) to increase engagement among younger users, particularly in primary and secondary school contexts. Such features are expected to enhance motivation, adherence, and the educational impact of postural training. Thus, qualitative insights not only confirm usability but also point toward the system’s potential pedagogical relevance, which should be validated with objective educational outcomes in future trials.

### Broader Applicability and Future Directions

Moreover, although this study did not include professional educators or clinicians as participants, the students’ perspective already points toward broader applicability. Future studies could involve instructors, physical therapists, or school teachers to explore how the system can be integrated into formal curricula or used in clinical decision-making. There is growing interest in using wearable technology in both school-based [75] and outpatient rehabilitation [76] settings, and EduBack may offer a scalable, cost-effective option that meets usability standards in both domains.

In practical terms, EduBack could be integrated into physical education curricula through teacher-guided sessions, where students receive real-time feedback while performing standardized postural exercises. The system may also be incorporated into modules on ergonomics or injury prevention, allowing teachers to demonstrate correct lifting techniques or seated posture with immediate visual reinforcement. Adolescents could use the app independently under supervision, while younger children would benefit from structured, teacher-mediated activities. These scenarios illustrate how EduBack can complement existing educational practices, providing an interactive tool that supports awareness, prevention, and the acquisition of healthy motor habits within the classroom environment.

EduBack was designed primarily for educational environments, with physical education teachers expected to guide its application. Adolescents may be able to use the system independently, while younger children would require teacher supervision. Clarifying these user profiles is essential to ensure appropriate implementation and will be a key focus of future research.

Future research should also focus on multisite evaluations involving diverse populations and formal educational settings, possibly incorporating mixed method approaches to assess user engagement, learning outcomes, and behavioral adherence. It would also be beneficial to test EduBack in younger populations (eg, primary or secondary school students) and in clinical training contexts, where postural education is part of professional development.

### Limitations

This study has several limitations that should be considered when interpreting the findings. First, the sample size was small and limited to university students, which may affect generalizability and highlights the need to test EduBack in younger and more diverse populations. Second, technical performance was evaluated in controlled academic settings; future studies should assess robustness in less stable environments such as classrooms or homes. Third, while the SUS provided a validated measure of usability, future studies could complement it with domain-specific instruments, such as the mobile health App Usability Questionnaire, to capture nuances related to health behavior change and long-term adherence. Finally, the study focused on short-term interactions; longitudinal research is needed to evaluate sustained use, user satisfaction, and potential impacts on posture-related outcomes.

### Conclusions

Beyond the usability and technical outcomes, a key contribution of this study is the creation of EduBack itself, a novel educational tool that integrates wearable sensors and real-time biofeedback for postural training. EduBack demonstrates strong usability and technical reliability as a tool for posture-related education. It fills an important gap in the current landscape of mobile health apps by providing real-time, interactive biofeedback specifically tailored for learning rather than therapy. The system offers a promising foundation for future educational interventions in schools, universities, and clinical environments aimed at promoting postural health, awareness, and motor competence.

## Abbreviations

IMU: inertial measurement unit
LBP: low back pain
SUS: system usability scale
